# Assessment of Rural-Urban Differences in Health Care Use and Survival Among Medicare Beneficiaries With Alzheimer Disease and Related Dementia

**DOI:** 10.1001/jamanetworkopen.2020.22111

**Published:** 2020-10-22

**Authors:** Momotazur Rahman, Elizabeth M. White, Kali S. Thomas, Eric Jutkowitz

**Affiliations:** 1 Department of Health Services, Policy and Practice, Brown University School of Public Health, Providence, Rhode Island

## Abstract

**Importance:**

There is poor understanding as to how survival and health care use varies among older adults living with Alzheimer disease and related dementia (ADRD) in rural vs urban areas of the United States.

**Objective:**

To describe survival and trajectories of hospital, hospice, nursing home, and home health care use among rural and urban Medicare beneficiaries with ADRD in the 6 years after diagnosis.

**Design, Setting, and Participants:**

This retrospective cohort study linked Medicare claims data from January 1, 2009, to December 31, 2016, with nursing home and home health assessment data from all US counties. A total of 555 333 Medicare fee-for-service beneficiaries newly diagnosed with ADRD in 2010 were included. A total of 424 561 individuals (76.5%) resided in metropolitan counties, 75 001 (13.5%) in micropolitan counties, and 55 771 (10.0%) in rural counties.

**Exposures:**

Rurality of beneficiary’s county of residence.

**Main Outcomes and Measures:**

Number of days survived after initial ADRD diagnosis; percent of survived days per month spent in the hospital, hospice nursing home, community with home health care services, and community without home health care services.

**Results:**

A total of 555 333 Medicare beneficiaries (mean [SD] age, 82.0 [7.5] years; 345 294 women [62.2%]; 480 286 White [86.5%]) were evaluated. Compared with metropolitan county residents, rural beneficiaries were younger (mean [SD] age, 81.6 [7.6] vs 82.1 [7.5] years), were less likely to be women (34 100 [61.1%] vs 264 688 [62.3%]), were more likely to be White (50 886 [91.2%] vs 361 205 [85.1%]) and Medicaid-eligible (14 264 [25.6%] vs 71 656 [16.9%]), and had fewer preexisting chronic conditions (mean [SD], 6.9 [2.8] vs 7.4 [2.9]). Medicare beneficiaries residing in metropolitan counties survived a mean (SD) of 1183.5 (826.0) days after diagnosis. Adjusting for individual demographic and clinical characteristics, rural and micropolitan county residents survived approximately 1.5 months less than metropolitan residents. The adjusted share of survived days spent in nursing homes was 5.7 (95% CI, 4.0-7.5) percentage points higher for rural vs metropolitan residents. The adjusted share of days in hospitals was 0.7 (95% CI, –0.9 to –0.4) percentage points lower, and the share of days in community without home health care was 4.6 (95% CI, –6.1 to –3.1) percentage points lower for rural vs metropolitan county residents. There were no statistically significant differences in home health or hospice use. Similar patterns were found for micropolitan vs metropolitan residents as for rural vs metropolitan residents, although the magnitude of the differences were smaller. Differences in time spent in community and nursing homes between rural vs metropolitan beneficiaries became more pronounced with further time from diagnosis.

**Conclusions and Relevance:**

Study results suggest that, after diagnosis, rural Medicare beneficiaries with ADRD spend more time in nursing homes and less time in the community, receive less home health care, and have shorter survival than their urban counterparts.

## Key Points

**Question:**

How do health care use and survival differ between older Americans with Alzheimer disease and related dementia (ADRD) in rural vs urban counties?

**Findings:**

This cohort study of 555 333 Medicare beneficiaries with ADRD diagnosed in 2010 found that rural county residents experienced lower survival and fewer hospital inpatient days than urban county residents in the 6 years after diagnosis. Compared with urban county residents, rural residents spent more days in nursing homes, fewer days in the community, and a similar amount of time in home health and hospice.

**Meaning:**

Study findings suggest that after diagnosis, rural Medicare beneficiaries with ADRD spend more time in nursing homes, less time in the community, and have shorter survival time than their urban counterparts.

## Teaser

This cohort study uses Medicare claims data from nursing home and home health assessments from all US counties to investigate how health care use and survival compares between individuals with Alzheimer disease and related dementia in rural vs urban settings.

## Introduction

Alzheimer disease and related dementia (ADRD) affects roughly 9% of adults older than age 65 years, causing declines in cognition and the ability to perform daily activities.^1^ The number of Americans living with Alzheimer disease, the most common cause of dementia, is projected to increase in the coming years from 5.8 million in 2019 to almost 14 million by 2050.^2^ Rural populations are, on average, older than urban populations and have higher rates of chronic conditions, such as hypertension, diabetes, obesity, hyperlipidemia, traumatic brain injury, and depression. Rural populations also have higher rates of alcoholism and tobacco use and lower levels of formal educational attainment than urban populations.^3,4,5^ Combined, these risk factors suggest that rural communities may be disproportionately affected by ADRD.

Besides individual risk factors, rural residents with ADRD may also have more limited access to health care and community-based long-term care services than their urban counterparts. As individuals with ADRD progress into more advanced disease stages, they increasingly rely on family and friend care partners for assistance with daily activities.^6,7^ Older adults in rural areas are generally more likely to live with family than those in urban areas^8^; however, those who live alone may live far away from family and other caregivers.^9^ Nursing homes have historically been the dominant site of care for people with severe dementia^10,11^; however, these individuals are increasingly remaining in the community with the support of home health aides, adult day programs, and other community-based long-term care services.^12,13^ There is large regional variation in the availability of nursing homes and community-based services.^14,15^ Rural county residents are more likely to receive postacute care in nursing homes and less likely to receive home health care than urban county residents.^16^

Much of what we know about rural-urban disparities in dementia care has come from small descriptive studies of specific rural communities with small sample sizes.^17^ Lacking are detailed longitudinal studies that explore variation in health care use and outcomes between rural and urban communities across the entire country. The absence of such information hinders efficient and informed care planning and attenuates policy efforts to ameliorate rural-urban disparities in health outcomes. To fill this gap in the literature, we described survival and use of hospital, nursing home, and home health care among rural and urban Medicare beneficiaries with ADRD. Using Medicare claims and assessment data, we compared trajectories of health care use in the 6 years after ADRD diagnosis among Medicare beneficiaries diagnosed in 2010.

## Methods

Brown University’s institutional review board approved the study protocol and waived the requirement for participant informed consent because of the infeasibility of acquiring consent in claims data. Medicare claims with nursing home and home health assessment data from January 1, 2009, to December 31, 2016, were analyzed. We linked 100% Medicare claims and assessment data to identify fee-for-service Medicare beneficiaries newly diagnosed with ADRD in 2010 and assessed their health care use and outcomes in the subsequent 72 months. This study followed the Strengthening the Reporting of Observational Studies in Epidemiology (STROBE) reporting guideline.

### Data Sources

Our primary sources of individual-level data were the Master Beneficiary Summary File and Medicare claims data. The Master Beneficiary Summary File includes individuals’ state and county of residence, demographic characteristics, date of death, and monthly managed care and Medicaid eligibility indicators. We also used the Chronic Condition Warehouse section that includes annual flags and first date of diagnosis to identify ADRD diagnoses. The Medicare claims data include Medicare claims for inpatient, skilled nursing facility, home health agency, hospice, and outpatient services. We supplemented claims data with nursing home and home health patient assessment data to better ascertain care use. All nursing homes (Minimum Data Set) and home health agencies (Outcome and Assessment Information Set) certified to accept Medicare and Medicaid payment are required to complete assessments on all postacute and long-term patients. County-level data were obtained from the Area Health Resource File. We also used zip code–level social deprivation index data compiled by the Robert Graham Center.

By combining these data, we have built a Residential History File, which is a per-person chronological history of health service use and location of service.^18,19,20^ To create the Residential History File, use information from claims, the Outcome and Assessment Information Set, and the Minimum Data Set are filled into the calendar days according to the following hierarchy: inpatient claims, skilled nursing facility claims, home health claims, hospice claims, Minimum Data Set projected episodes, Outcome and Assessment Information Set projected home health episodes, and continued nursing home stay indicated by outpatient claims with nursing home location. Because hospice can be provided in a variety of settings (at home, in nursing home, as inpatient hospice), we assigned the relevant episodes to hospice. We used the Residential History File to create several control and outcome variables.

### Identification of ADRD

We used the Medicare Master Beneficiary Summary File Chronic Condition Warehouse (CCW) year-end flag for Alzheimer Disease, Related Disorders, or Senile Dementia to identify fee-for-service Medicare beneficiaries with an ADRD diagnosis in a given calendar year. The CCW algorithm captures 24 *International Classification of Diseases, Ninth Revision (ICD-9)* codes for dementia present within a 3-year lookback on 1 or more inpatient, nursing home, home health, outpatient, or carrier claim. This algorithm had a sensitivity of 0.85 and specificity of 0.89 when compared with clinically diagnosed cases of dementia in the Aging, Demographics, and Memory Study.^21^ The CCW file also includes the first date the beneficiary met claims criteria for ADRD, dating back to January 1, 1999. We used this initial flag as the date of diagnosis.

### Cohort

Our cohort included all Medicare beneficiaries who had an ADRD flag with a date of diagnosis in 2010. By construction of the CCW data set, all of these beneficiaries continued having the flag in the subsequent years. These beneficiaries were enrolled in fee-for-services Medicare in all years of the study.

### Metropolitan, Micropolitan, and Rural

We used the Medicare Master Beneficiary Summary File to determine each Medicare beneficiary’s county of residence. We then used the Area Resource File to determine the rurality of a beneficiary’s county: metropolitan (rural-urban continuum codes 1-3), micropolitan (rural-urban continuum codes 4-7), and rural (rural-urban continuum codes 8-9).^22,23,24,25,26,27^

### Outcomes

We examined beneficiaries’ survival and health care use for 72 months post-ADRD diagnosis. Studies indicate that adults aged 65 years and older survive an average of 4 to 8 years after diagnosis, yet some live as long as 20 years.^28,29,30^ Our first outcome is the number of days survived after an ADRD diagnosis. Second, among those who were alive in a given follow-up month, we examined the share of days in that month spent in 1 of the 5 mutually exclusive locations: (1) inpatient hospital, (2) nursing home, (3) community with home health care services, (4) hospice, and (5) community without home health care services. We also aggregated these outcomes to measure overall care use over the first 72 months after ADRD diagnosis.

### Covariates

We obtained beneficiary demographic characteristics (age, sex, race/ethnicity, and Medicaid eligibility) from the Medicare Beneficiary Summary File. We classified beneficiaries’ comorbidities using 2 measures: (1) a count of chronic and potentially disabling conditions identified in the CCW file for the year before ADRD diagnosis and (2) a hierarchical chronic condition score derived from *ICD-9* codes reported on all Part A claims during the year before ADRD diagnosis. We also computed summary measures of each beneficiary’s health care use in the prior year including the number of inpatient hospital admissions, days in a nursing home, and a count of home health assessments.^14,31^ To characterize beneficiaries’ neighborhoods, we determined the share of Medicare beneficiaries eligible for Medicaid and the share of beneficiaries enrolled in Medicare Advantage for each beneficiary’s zip code. We also included zip code level of the social deprivation index, which is based on neighborhood social determinants of health, including demographic composition, education and income levels, etc.^32^

### Statistical Analysis

We describe the characteristics of Medicare beneficiaries residing in metropolitan, micropolitan, and rural counties who had new ADRD diagnoses in 2010. For the outcome analyses, we started by comparing survival and health care use outcomes over the 72 months after diagnosis. We first calculated unadjusted differences in outcomes of micropolitan and rural county residents compared with metropolitan county residents. We also estimated the risk-adjusted difference using a linear regression model of an outcome onto indicators for micropolitan and rural counties and the covariates.

Comparisons between rural and urban residents living with ADRD are likely confounded by differences in health status at the time of diagnosis. In general, we expected the rural ADRD incident population to be sicker at diagnosis because of higher prevalence of chronic conditions among rural populations.^3,4,5^ Additionally, urban counties compared to rural counties have a greater concentration of geriatricians and other specialists to diagnose and treat ADRD. Therefore, urban county residents may be diagnosed earlier in the disease course. However, higher life expectancy in urban counties^27^ can result in a sicker ADRD incident population in urban areas. Because of a higher life expectancy, for some urban beneficiaries with ADRD, had they been residing in rural counties, they would have died before being diagnosed with ADRD. Thus, higher life expectancy in urban counties can cause sicker and older beneficiaries to be diagnosed with ADRD. We adjust for this difference by including a series of demographic characteristics, comorbidities, prior health care use data, and neighborhood characteristics to account for differences in health status and health care access.

To understand differences in health care use trajectories between rural and urban counties, we first plotted average outcomes experienced by metropolitan, micropolitan, and rural county residents over the 72 months after an ADRD diagnosis. We estimated the adjusted difference using a linear regression model of an outcome onto indicators for micropolitan and rural counties and the covariates separately for each month. Thus, we estimated 72 models for each outcome. We used this estimation specification following previous articles on trajectory analysis based on the Residential History File.^19,33^ Estimating a separate model for each month allows risk factors, such as age at diagnosis, to have varying effects on outcomes over time. Similarly, we did not update the risk adjusting variables for each follow-up month because we hypothesize that these are affected by rurality and therefore endogenously determined. We then plotted the coefficients (along with the 95% CIs) of micropolitan and rural indicators over the 72 months.

Because there are large differences in characteristics between rural and urban residents, we performed a robustness analysis comparing metropolitan, micropolitan, and rural county residents who are observed to be similar in terms of propensity score. This analysis involved the following steps. First, we estimated a multinomial logit model of rurality (metropolitan vs micropolitan vs rural) onto all covariates. Second, we predicted the likelihood of living in a metropolitan county in 2010 (propensity score) and identified beneficiaries in deciles of this predicted likelihood. Third, we estimated the risk-adjusted difference in outcomes of micropolitan and rural county residents compared with metropolitan county residents separately for each decile group.

We conducted 2 additional robustness checks using various beneficiary subgroups. First, because urban Medicare beneficiaries are more likely to enroll in Medicare Advantage than rural beneficiaries, we estimated the risk-adjusted differences in outcomes of micropolitan and rural county residents compared with metropolitan county residents separately for zip codes with different levels of Medicare Advantage penetration. Second, because there are significant socioeconomic disparities between rural and urban areas, we estimated differences in outcomes separately for quartiles of zip codes with different levels of social deprivation index.

All analyses were conducted using Stata software, version 16 (StataCorp LLC) from October 1, 2019, to July 15, 2020. The 95% CIs were based on robust SEs clustered by state. *P* values were 2-sided, and a significance level was set at .05. We clustered at the state level to account for beneficiaries within a given state being exposed to the same state policies affecting long-term care, such as Certificate of Need laws and Medicaid waivers for Home and Community-based Services.

## Results

The eTable in the Supplement summarizes the selection process, which yielded 555 333 (mean [SD] age, 82.0 [7.5] years; 345 294 women [62.2%]; 480 286 White [86.5%]) Medicare fee-for-service beneficiaries who were newly diagnosed with ADRD in 2010. A total of 424 561 (76.5%) of these individuals were in metropolitan counties, 75 001 (13.5%) were in micropolitan counties, and 55 771 (10.0%) were in rural counties. Compared with metropolitan county residents, rural beneficiaries were younger (mean [SD] age, 81.6 [7.6] vs 82.1 [7.5] years), were less likely to be women (34 100 [61.1%] vs 264 688 [62.3%]), were more likely to be White (50 886 [91.2%] vs 361 205 [85.1%]) and Medicaid-eligible (14 264 [25.6%] vs 71 656 [16.9%]), and had fewer preexisting chronic conditions (mean [SD], 6.9 [2.8] vs 7.4 [2.9]). Table 1 presents characteristics of beneficiaries with ADRD during the month of initial diagnosis. The share of individuals dually eligible for Medicaid varies widely across counties, with 71 656 (16.9%) in metropolitan, 16 382 (21.8%) in micropolitan, and 14 264 (25.6%) in rural counties. Yet, the hierarchical chronic condition score derived from Part A claims was similar across all counties (mean [SD], 0.9 [0.7] in rural counties vs 0.9 [0.8] in metropolitan counties). Rural beneficiaries spent more time in nursing homes, hospitals, and with home health care services in the year before their ADRD diagnosis compared with beneficiaries residing in metropolitan and micropolitan counties. The neighborhood social deprivation score was higher among residents in rural (mean [SD], 50.3 [21.5]) and micropolitan (mean [SD], 53.3 [22.0]) counties than those in metropolitan counties (mean [SD], 46.3 [28.6]). Although most of these differences were small in magnitude, all of them were statistically significant with *P* < .001.

**Table 1.** Characteristics of Medicare Beneficiaries Newly Diagnosed With ADRD in 2010, Residing in Metropolitan, Micropolitan, and Rural Counties

Variable^a^	Mean (SD)^b^		
	Metropolitan (n = 424 561)	Micropolitan (n = 75 001)	Rural (n = 55 771)
Age, y	82.1 (7.5)	81.6 (7.5)	81.6 (7.6)
Women, No. (%)	264 688 (62.3)	46 506 (62.0)	34 100 (61.1)
White, No. (%)	361 205 (85.1)	68 195 (90.9)	50 886 (91.2)
Black, No. (%)	33 610 (7.9)	4047 (5.4)	3138 (5.6)
Hispanic, No. (%)	18 684 (4.4)	1652 (2.2)	912 (1.6)
Other race, No. (%)^c^	11 037 (2.6)	1107 (1.5)	835 (1.5)
Dual eligible, No. (%)	71 656 (16.9)	16 382 (21.8)	14 264 (25.6)
Chronic conditions			
Chronic conditions, No.^d^	7.4 (2.9)	7.0 (2.8)	6.9 (2.8)
Other chronic or potentially disabling conditions, No.^d^	1.6 (1.5)	1.5 (1.5)	1.5 (1.5)
HCC score, No.^e^	0.9 (0.8)	0.9 (0.7)	0.9 (0.7)
Health care use in the year before diagnosis			
Hospitalizations, No.	0.5 (1.1)	0.5 (1.1)	0.6 (1.3)
Nursing home days, No.	13.3 (57.1)	18.6 (70.5)	21.3 (75.6)
Home health assessments, No.	0.51 (1.3)	0.52 (1.4)	0.57 (1.6)
Residential zip code characteristics			
Medicare beneficiaries in Medicare Advantage, %	21.3 (12.5)	13.4 (9.5)	11.4 (8.3)
Medicare beneficiaries who are dual eligible, %	15.9 (10.4)	18.3 (8.3)	20.2 (9.3)
Social deprivation score	46.3 (28.6)	53.3 (22.0)	50.3 (21.5)

Abbreviations: ADRD, Alzheimer disease and related dementia; HCC, hierarchical chronic condition.

^a^ For all variables, the mean values for micropolitan and rural counties are statistically different from the mean values for metropolitan counties with *P* values <.001.

^b^ Values are expressed as mean (SD) unless otherwise specified.

^c^ Includes Asian, North American Native, and Other (as coded in the Medicare benificiary summary file).

^d^ As reported in the Chronic Conditions Warehouse in the year before ADRD diagnosis.

^e^ Hierarchical Chronic Condition score derived from Part A Medicare claims in the year before ADRD diagnosis.

Table 2 presents aggregated outcomes for individuals in the 72 months following an ADRD diagnosis. The average number of days survived after diagnosis were effectively the same for beneficiaries in metropolitan, micropolitan, and rural counties (mean [SD] of 1183.5 [826.0] days after diagnosis for metropolitan counties, with difference from rural counties statistically insignificant). Similarly, for all counties, 27% of the beneficiaries survived the entire follow-up period of 72 months (eFigure 1 in the Supplement plots the percentage of beneficiaries alive at each follow-up month). Of these survived days, metropolitan beneficiaries spent 64.2% of days in the community without home health care, 3.7% of days in the community with home health care, 20.4% of days in nursing homes, 5.5% days with hospice care, and 6% of days in hospitals. Although the number of unadjusted days survived was the same for rural and metropolitan residents, adjusting for individual characteristics, metropolitan county residents survived about 1.5 months longer than micropolitan and rural residents. The adjusted share of survived days spent in the community without home health care was 2.8 percentage points lower (95% CI, –4.1 to –1.6) for micropolitan vs metropolitan county residents and 4.7 percentage points lower (95% CI, –6.2 to –3.2) for rural vs metropolitan county residents. Conversely, the share of survived days spent in nursing homes was 5.7 percentage points higher (95% CI, 4.0-7.5) for rural vs metropolitan residents and 3.7 percentage points higher (95% CI, 2.3-5.0) for micropolitan vs metropolitan residents. Rural and micropolitan residents also spent a significantly lower share of days in hospitals compared with metropolitan residents.

**Table 2.** Number of Days Residing in Different Settings in 6 Years After ADRD Diagnosis

Outcome	Metropolitan counties	Relative to metropolitan counties			
		Unadjusted difference		Adjusted difference^**a**^	
		Micropolitan	Rural	Micropolitan	Rural
Days survived, No.	1183.5	−9.7^b^	−4.1	−41.5 (−61.8 to −21.3)^c^	−47.1 (−72.6 to −21.5)^b^
Individuals survived 72 mo after ADRD diagnosis, %	27.4	−0.8^c^	−0.6^c^	−2.4 (−3.8 to −1.0)^c^	−2.5 (−4.0 to −1.9)^c^
Survived days in community without home health care services, %	64.2	−3.3^c^	−5.6^c^	−3.0 (−4.1 to −1.6)^c^	−4.7 (−6.1 to −3.1)^c^
Survived days in community with home health care services, %	3.7	−0.5^c^	−0.5^c^	−0.2 (−0.5 to 0.2)	−0.2 (−0.5 to 0.2)
Survived days in nursing home, %	20.4	5.1^c^	8.0^c^	3.7 (2.3 to 5.0)^c^	5.7 (4.0 to 7.5)^c^
Survived days with hospice care, %	6.3	−0.5^c^	−0.9^c^	−0.1 (−0.6 to 0.4)	−0.3 (−0.9 to 0.3)
Survived days in hospital, %	5.5	−0.8^c^	−1.0^c^	−0.5 (−0.7 to −0.2)^c^	−0.7 (−0.9 to −0.4)^c^

Abbreviation: ADRD, Alzheimer disease and related dementia; OLS, ordinary least squares.

^a^ Adjusted differences are estimated running OLS regressions of outcomes onto indicators for micropolitan and rural counties (metro counties are the reference category) and patient characteristics reported in Table 1. Confidence intervals and *P* values are obtained clustering errors by state.

^b^ *P* < .01.

^c^ *P* < .001.

**Figure 1.**
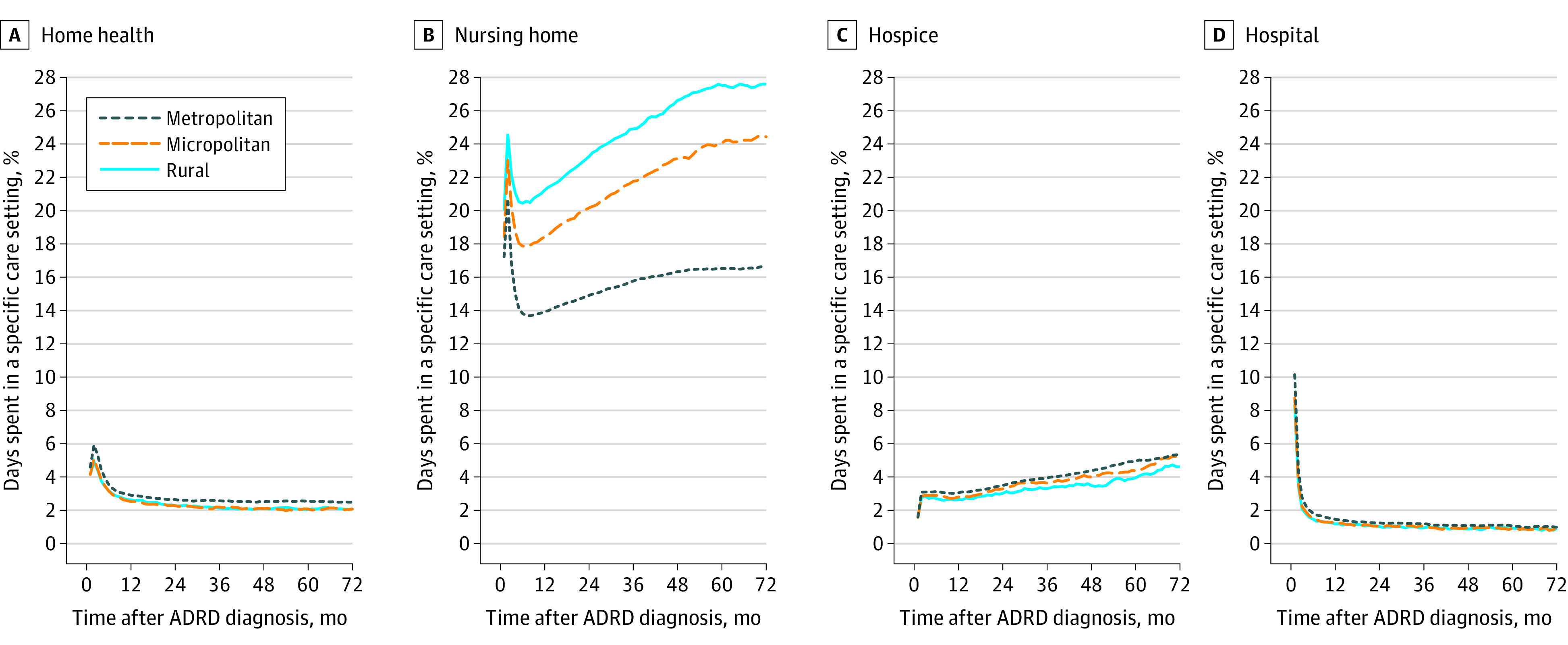
Outcomes in 72 Months After ADRD Diagnosis for Medicare Beneficiaries Residing in Metropolitan, Micropolitan, and Rural Counties The plots for days in community are not presented because of differences in scale. The number of survived days in the community for any month is equal to 100 minus the summation of values in 4 settings and have been presented in eFigure 2 in the Supplement. ADRD indicates Alzheimer disease and related dementia.

Figure 1 plots care use trajectories over the 72 months after ADRD diagnosis. Beneficiaries were in hospitals for approximately 10% of days in the first month following an ADRD diagnosis, and this rate dropped to 2% in the following month. Approximately 41% of metropolitan residents and 39% of rural and micropolitan county residents had an inpatient stay in the first month of ADRD diagnosis. We also observed a spike in nursing home and home health care use on the second month, reflecting postacute care episodes. Across the 72 months, metropolitan county residents spent fewer days in nursing homes and more days with home health and hospice care compared with rural and micropolitan county residents. The differences in nursing home use for metropolitan vs rural and micropolitan residents become larger the further the time from ADRD diagnosis. For example, 1 year after diagnosis, the share of survived days spent in nursing homes was 14%, 18%, and 21% among metropolitan, micropolitan, and rural county residents, respectively. By 72 months, these rates were 17%, 24%, and 28%, respectively.

**Figure 2.**
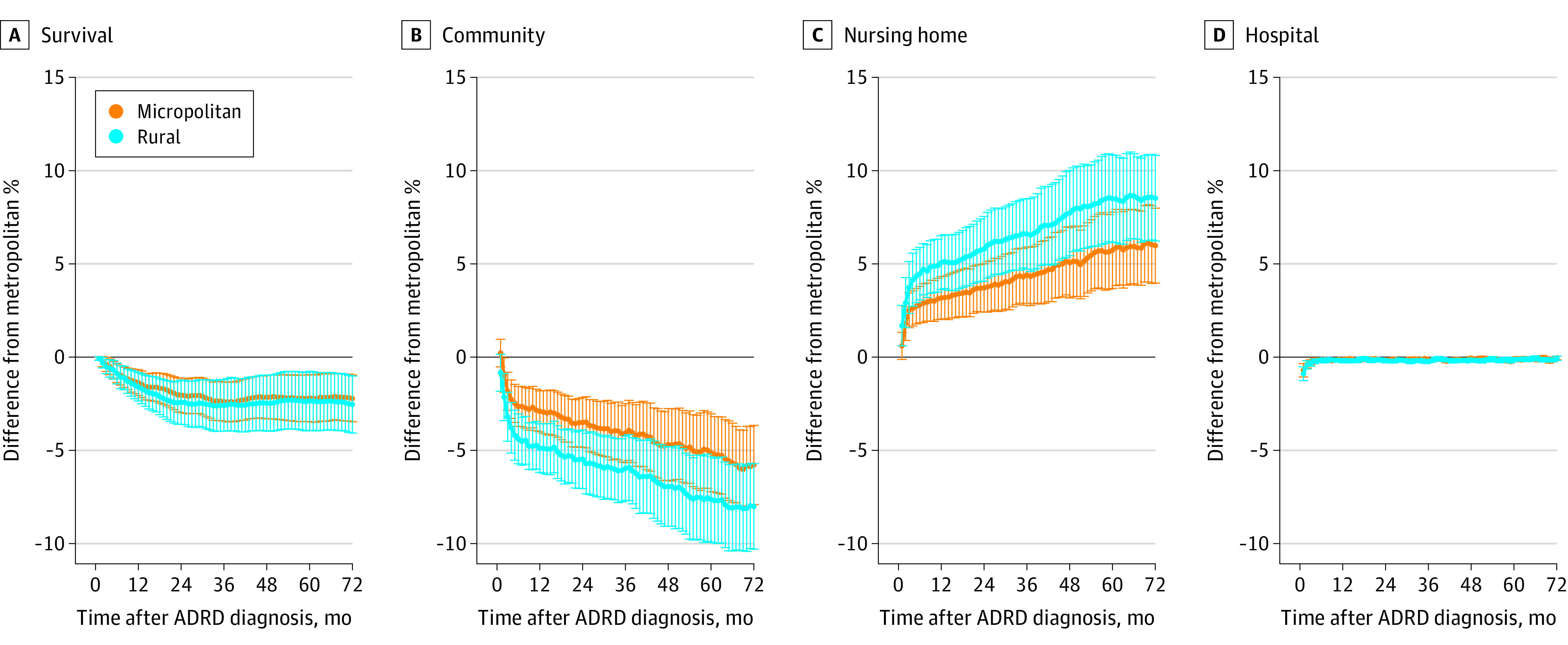
Difference in Outcomes for Medicare Beneficiaries With ADRD Residing in Micropolitan and Rural Counties Relative to Those in Metropolitan Counties Adjusted differences are estimated running OLS regressions of outcomes onto indicators for micropolitan and rural counties (metro counties are the reference category) and patient characteristics reported in Table 1. A regression model was estimated for each follow-up month of a specific outcome. 95% CIs are obtained clustering errors by state. Panel B plots community without home health care. Estimates for home health and hospice care are not statistically significant for any month and have been presented in eFigure 3 in the Supplement. ADRD indicates Alzheimer disease and related dementia; OLS, ordinary least squares.

Figure 2 plots the adjusted differences in outcomes for residents of micropolitan and rural counties compared with those of metropolitan counties. Residents in metropolitan counties had more community days without home health care compared with residents in rural and micropolitan counties. Hospital use in both rural and micropolitan counties was lower than in metropolitan counties. These differences were higher during the first few months after diagnosis (for example, approximately 0.6 percentage points in the second month) and became fairly small (0.1 percentage point in month 72 with *P* value <.01) during the later follow-up months. Differences in home health care use and hospice care use between rural and urban beneficiaries were not statistically significant. The largest observed differences between rural and micropolitan vs metropolitan residents was in nursing home use. By 72 months, nursing home use rates were 8.5 percentage points higher in rural counties and 6.0 percentage points higher in micropolitan counties compared with metropolitan counties.

**Figure 3.**
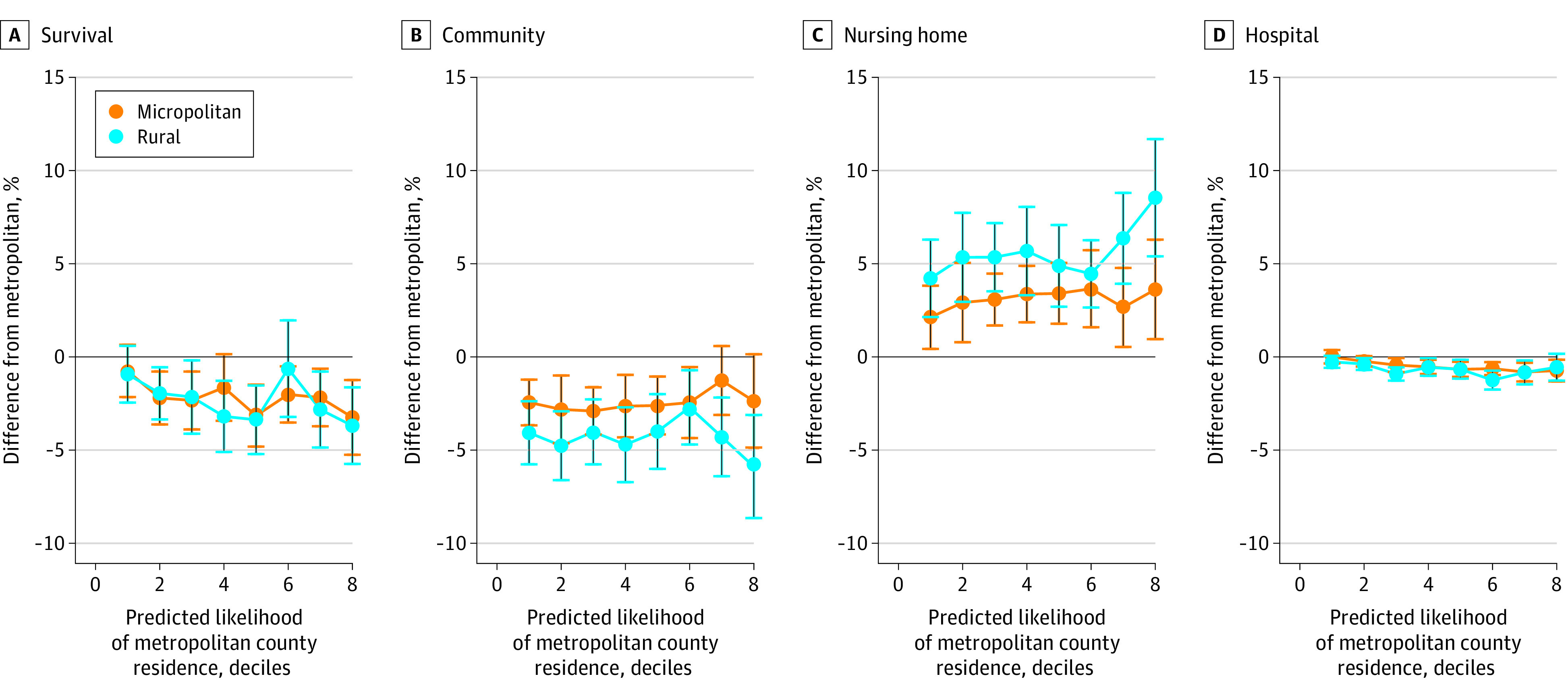
Share of Survived Days Spent in Different Care Settings Among Newly Diagnosed ADRD Patients as a Function of Predicted Likelihood of Metropolitan County Residence The regression results to predict this likelihood of metropolitan county residence are presented in eTable in the Supplement. The 9th and 10th decile groups are excluded from this plot because less than 3% of the rural residents belong to these 2 groups combined. Panel B plots community without home health care. Estimates for home health and hospice care are not statistically significant and have been presented in eFigure 4 in the Supplement. ADRD indicates Alzheimer disease and related dementia.

Figure 3 presents the results of our robustness analyses with the risk-adjusted difference in outcomes for each decile group of beneficiaries based on likelihood of metropolitan county residence. We did not examine the ninth and 10th decile groups because less than 3% of the rural residents belong to these 2 groups combined. Figure 3 shows consistent findings across all decile groups that rural and micropolitan county residents experienced higher nursing home use, fewer days in community, and lower hospital use than metropolitan county residents. eFigures 5 and 6 in the Supplement show the same patterns for different beneficiary subgroups based on counties’ Medicare Advantage penetration and neighborhood social deprivation index.

## Discussion

To our knowledge, this is the first national retrospective cohort study to assess rural-urban differences in survival and health care use trajectories over time for Medicare beneficiaries with ADRD. Very few studies have examined trajectories of care and outcomes of older adults with an ADRD diagnosis,^34,35,36^ and to our knowledge, none have been conducted on a national level. We found shorter risk-adjusted survival after diagnosis among rural county residents compared with metropolitan county residents. In addition, results suggest that rural residents spent more days in nursing homes and fewer days in the community than metropolitan residents, and these differences became more pronounced the further the time from diagnosis. We did not find any statistically significant differences in hospice or home health care use between beneficiaries in rural vs metropolitan counties.

Study results suggest that Medicare beneficiaries living with ADRD in rural and micropolitan counties survived about 1.5 months less than beneficiaries in metropolitan counties after adjustment for beneficiary and zip code characteristics. Importantly, the unadjusted rural-urban survival gap was small, and the adjusted results were mainly driven by metropolitan residents being older with more comorbidities at the time of diagnosis. This finding of metropolitan residents with ADRD being older and sicker at time of diagnosis was perplexing because it is well documented that rural populations have higher rates of chronic conditions.^3,4,5^ Although the share of the elderly population is higher in nonmetropolitan counties, the mean age of Medicare beneficiaries is lower in nonmetropolitan counties due to a lower life expectancy. Thus, a plausible explanation of our finding is that the higher life expectancy in metropolitan areas allows more individuals to be diagnosed with ADRD at more advanced ages.

Our results suggest that people living with ADRD in rural and micropolitan counties spent fewer days in hospitals than their urban counterparts. These findings are consistent with prior work showing fewer hospital readmissions among rural county residents.^16^ As rural hospitals increasingly close,^37^ older adults in rural communities may defer emergency department visits for more minor conditions for which metropolitan residents may be more likely to seek care.

In the 6 years after diagnosis, rural Medicare beneficiaries with ADRD spent approximately 1.3 times more days in nursing homes than metropolitan residents, adjusting for beneficiary demographic and clinical differences. Conversely, metropolitan residents were significantly more likely to remain in the community. This difference is likely driven by greater availability of home health care services,^16^ assisted living, and other community-based long-term care services such as adult day programs, home-delivered meals, transportation, and caregiver respite opportunities.^17,38^ The Affordable Care Act and state long-term care policies, particularly Medicaid 1915(c) waivers, have focused efforts on supporting individuals with ADRD to live in the community for as long as possible and avoid nursing home placement. An increase in the availability of community-based services in rural areas may reduce long-term nursing home care use. However, further work is needed to understand the costs and feasibility of such efforts. Other initiatives to improve the supply of primary care providers in rural areas, including removing scope of practice restrictions for nurse practitioners and physician assistants, may also help to improve access to care for older adults with ADRD to receive timely diagnoses and referrals to appropriate support services.^39,40^

Our findings suggest that there was a large proportion of beneficiaries who experienced an inpatient hospital stay on the month of diagnosis. This likely speaks to where people are being diagnosed. Of concern is that objective cognitive assessments conducted during an inpatient stay may be confounded by concurrent delirium, which has a high level of incidence among hospitalized older adults.^41^ Diagnostic criteria for dementia require that symptoms not be caused by delirium,^42^ and thus it is necessary for individuals to receive follow-up cognitive testing after hospitalization to confirm a dementia diagnosis. But for people with poor access to primary care, a hospitalization may be the only time they come in contact with medical professionals capable of making a diagnosis. Further work is needed to understand where and by whom people with ADRD are diagnosed, how this varies by rurality, and what the implications are for timely referral to appropriate support services.

The most important issue that can affect interpretation of our findings and presumed implications is unobserved difference in sickness between rural and urban beneficiaries. A potential explanation for fewer days in the community and shorter survival following diagnosis in nonmetropolitan counties is that nonmetropolitan residents may be diagnosed at later stages of disease compared with metropolitan residents, particularly given rural shortages of geriatricians and other geriatric specialists.^43^ Despite this shortage, Stensland et al^44^ found no significant differences between rural and urban beneficiaries in either the amount of health care received or satisfaction with access to care. Although we did not have data on disease stage at time of diagnosis, we think that this potential of length time bias in diagnosis of ADRD and severity at time of diagnosis is small for several reasons.

First, the share of elderly fee-for-service Medicare enrollees who were newly diagnosed with ADRD (diagnostic incidence rate) in 2010 were similar. with 2.9% in metropolitan counties and 2.7% in micropolitan and rural counties (eTable in the Supplement). Thus, the share of our cohort residing in metropolitan counties is roughly the same as the share of all elderly fee-for-service Medicare enrollees residing in metropolitan counties, and we probably do not have many missing nonmetropolitan ADRD incidence cases. Second, late diagnosis in nonmetropolitan counties would generally imply that that newly diagnosed ADRD population in nonmetropolitan counties would be older and sicker. In our cohort, nonmetropolitan county residents were younger and had fewer comorbidities though differences are arguably small. Third, if rural and micropolitan residents were diagnosed at later stages than metropolitan residents, we would expect the unadjusted survival gap between these groups to be large and the adjusted differences to be small. Yet, we found the opposite to be the case; unadjusted differences were relatively small, but adjusted differences were larger. We found shorter adjusted survival among nonmetropolitan residents because nonmetropolitan residents were younger and had fewer comorbidities during diagnosis and yet had similar survival compared with metropolitan county residents.

### Limitations

This study has some limitations. First, rurality is a continuous concept, which we stratified into 3 categories to simplify our presentation. We did not examine more detailed rurality categories, because our findings for micropolitan and rural counties were similar. Additionally, we assigned the rurality of each beneficiary’s county based on the year of diagnosis, and we did not assess change in residence county during the follow-up period. Second, we used claims data to identify ADRD, which is a less precise approach than has been undertaken in existing prospective cohort studies like the Aging, Demographics, and Memory Study in which participants must meet strict clinical diagnostic criteria. Medicare claims also do not provide information on dementia staging. We chose to use administrative data because it allowed us to look across all Medicare beneficiaries in the United States in order to understand rural-urban variation. Although Medicare claims have been found to have reasonable sensitivity and specificity for identifying dementia prevalence in a population,^21^ there have been no validation studies to our knowledge for using the incident claim for ADRD as the date of diagnosis. Thus, there is likely some estimation error in our approach, though that error would only vary by rurality if rural providers systematically bill for ADRD differently from urban providers (ie, by failing to submit ADRD codes for patients known to have dementia). We think this is unlikely. Finally, we cannot measure many beneficiary risk factors that may vary by rurality such as socioeconomics status, education, and access to social amenities. We attempted to address this issue by incorporating zip code level social deprivation index and share of Medicare beneficiaries dually eligible for Medicaid.

## Conclusions

Our findings suggest that rural Medicare beneficiaries with ADRD spend less time in the community than their urban counterparts. Rural Medicare beneficiaries with ADRD also appear to spend more time in nursing homes and experience shorter risk-adjusted survival and fewer hospitalizations than urban beneficiaries.

## Notes

Supplement. **eFigure 1.** Percentage of Days Survived in 72 Months Following ADRD Diagnosis for Medicare Beneficiaries Residing in Metropolitan, Micropolitan, and Rural Counties **eFigure 2.** Percentage of Survived Days Residing in Community Without Home Health Care Use in 72 Months Following ADRD Diagnosis for Medicare Beneficiaries Residing in Metropolitan, Micropolitan, and Rural Counties **eFigure 3.** Difference in Outcomes for Medicare Beneficiaries With ADRD Residing in Micropolitan and Rural Counties Relative to Those in Metropolitan Counties **eFigure 4.** Share of Survived Days Spent in Different Care Settings Among Newly Diagnosed ADRD Patients as a Function of Predicted Likelihood of Metropolitan County Residence **eFigure 5.** Difference in Outcomes for Medicare Beneficiaries With ADRD Residing in Micropolitan and Rural Counties Relative to Those in Metropolitan Counties by ZIP Codes With Different Medicare Advantage (MA) Penetration **eFigure 6.** Difference in Outcomes for Medicare Beneficiaries With ADRD Residing in Micropolitan and Rural Counties Relative to Those in Metropolitan Counties by ZIP Codes With Different Social Deprivation Index **eTable.** Cohort Selection Steps
