# Supplementary material for: Assessment of Rural-Urban Differences in Health Care Use and Survival Among Medicare Beneficiaries With Alzheimer Disease and Related Dementia
Source: JAMA Netw Open. 2020 Oct 22;3(10):e2022111. doi: 10.1001/jamanetworkopen.2020.22111 (PMC7582125; doi:10.1001/jamanetworkopen.2020.22111)
Supplement: Supplement. — eFigure 1. Percentage of Days Survived in 72 Months Following ADRD Diagnosis for Medicare Beneficiaries Residing in Metropolitan, Micropolitan, and Rural Counties eFigure 2. Percentage of Survived Days Residing in Community Without Home Health Care Use in 72 Months Following ADRD Diagnosis for Medicare Beneficiaries Residing in Metropolitan, Micropolitan, and Rural Counties eFigure 3. Difference in Outcomes for Medicare Beneficiaries With ADRD Residing in Micropolitan and Rural Counties Relative to Those in Metropolitan Counties eFigure 4. Share of Survived Days Spent in Different Care Settings Among Newly Diagnosed ADRD Patients as a Function of Predicted Likelihood of Metropolitan County Residence eFigure 5. Difference in Outcomes for Medicare Beneficiaries With ADRD Residing in Micropolitan and Rural Counties Relative to Those in Metropolitan Counties by ZIP Codes With Different Medicare Advantage (MA) Penetration eFigure 6. Difference in Outcomes for Medicare Beneficiaries With ADRD Residing in Micropolitan and Rural Counties Relative to Those in Metropolitan Counties by ZIP Codes With Different Social Deprivation Index eTable. Cohort Selection Steps [file jamanetwopen-e2022111-s001.pdf]

Supplementary Online Content

Rahman M, White EM, Thomas KS, Jutkowitz E. Assessment of rural-urban differences in health care use and survival among Medicare beneficiaries with Alzheimer disease and related dementia. *JAMA Netw Open*. 2020;3(10):e2022111. doi:10.1001/jamanetworkopen.2020.22111

eFigure 1. Percentage of Days Survived in 72 Months Following ADRD Diagnosis for Medicare Beneficiaries Residing in Metropolitan, Micropolitan, and Rural Counties

eFigure 2. Percentage of Survived Days Residing in Community Without Home Health Care Use in 72 Months Following ADRD Diagnosis for Medicare Beneficiaries Residing in Metropolitan, Micropolitan, and Rural Counties

eFigure 3. Difference in Outcomes for Medicare Beneficiaries With ADRD Residing in Micropolitan and Rural Counties Relative to Those in Metropolitan Counties

eFigure 4. Share of Survived Days Spent in Different Care Settings Among Newly Diagnosed ADRD Patients as a Function of Predicted Likelihood of Metropolitan County Residence

eFigure 5. Difference in Outcomes for Medicare Beneficiaries With ADRD Residing in Micropolitan and Rural Counties Relative to Those in Metropolitan Counties by Zip Codes With Different Medicare Advantage (MA) Penetration

eFigure 6. Difference in Outcomes for Medicare Beneficiaries With ADRD Residing in Micropolitan and Rural Counties Relative to Those in Metropolitan Counties by Zip Codes With Different Social Deprivation Index

eTable. Cohort Selection Steps

This supplementary material has been provided by the authors to give readers additional information about their work.

© 2020 Rahman M et al. *JAMA Network Open.*

eFigure 1. Percentage of Days Survived in 72 Months Following ADRD Diagnosis for Medicare Beneficiaries Residing in Metropolitan, Micropolitan, and Rural Counties

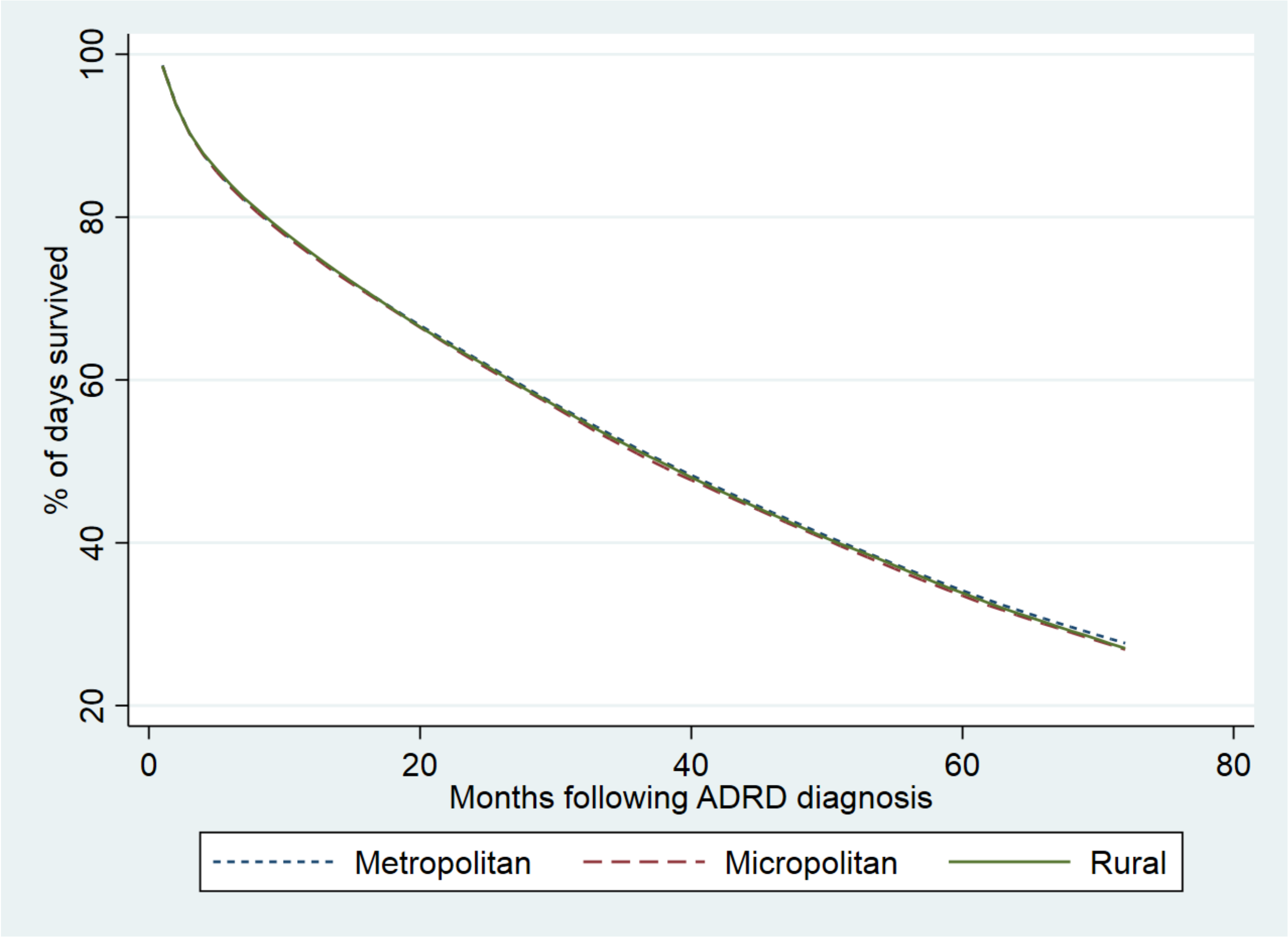

© 2020 Rahman M et al. *JAMA Network Open.*

eFigure 2. Percentage of Survived Days Residing in Community Without Home Health Care Use in 72 Months Following ADRD Diagnosis for Medicare Beneficiaries Residing in Metropolitan, Micropolitan, and Rural Counties

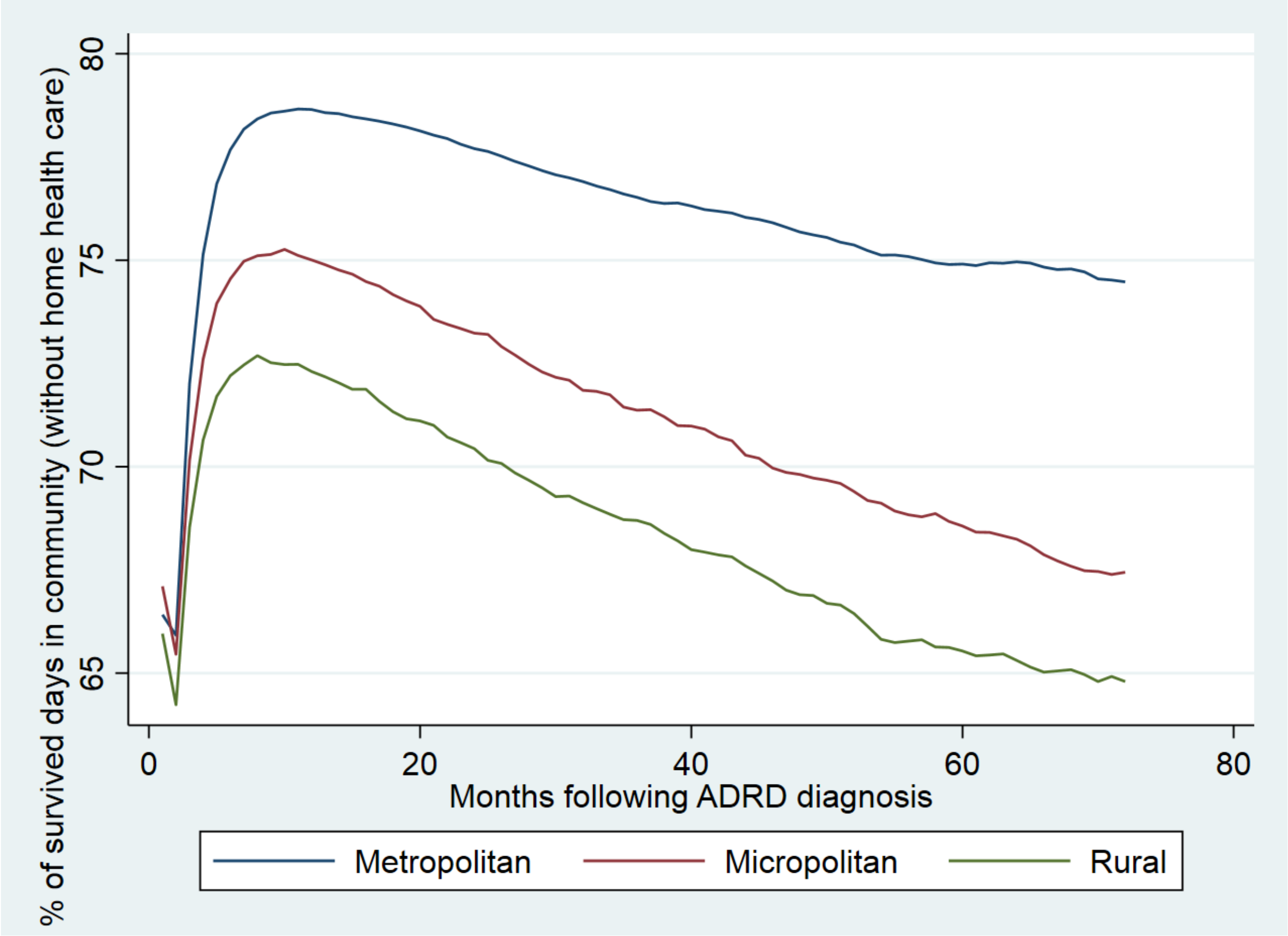

Note: This is the complementary figure of Figure 1 in the manuscript

© 2020 Rahman M et al. *JAMA Network Open.*

eFigure 3. Difference in Outcomes for Medicare Beneficiaries With ADRD Residing in Micropolitan and Rural Counties Relative to Those in Metropolitan Counties

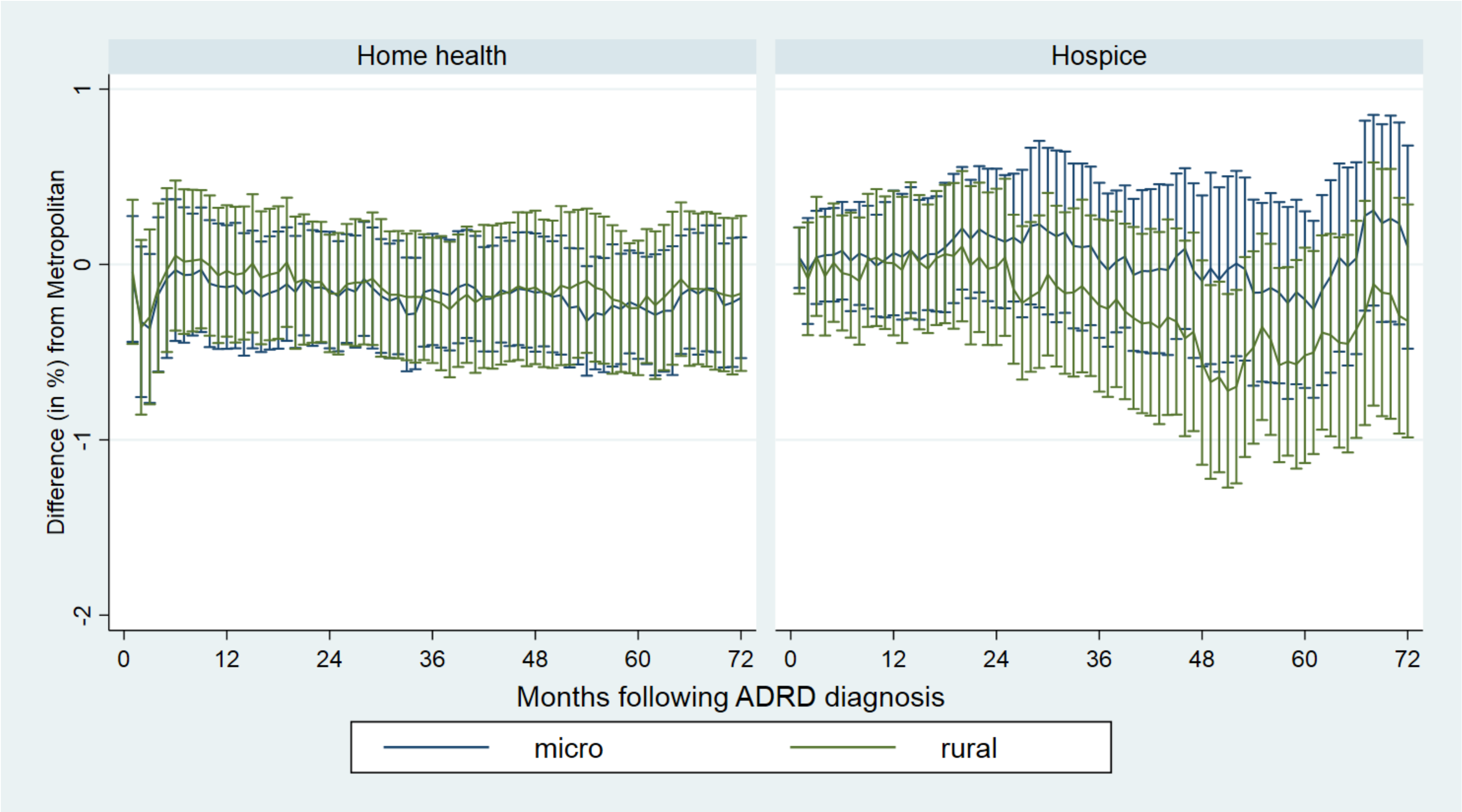

Note: This is the complementary figure of Figure 2 in the manuscript

© 2020 Rahman M et al. *JAMA Network Open.*

eFigure 4. Share of Survived Days Spent in Different Care Settings Among Newly Diagnosed ADRD Patients as a Function of Predicted Likelihood of Metropolitan County Residence

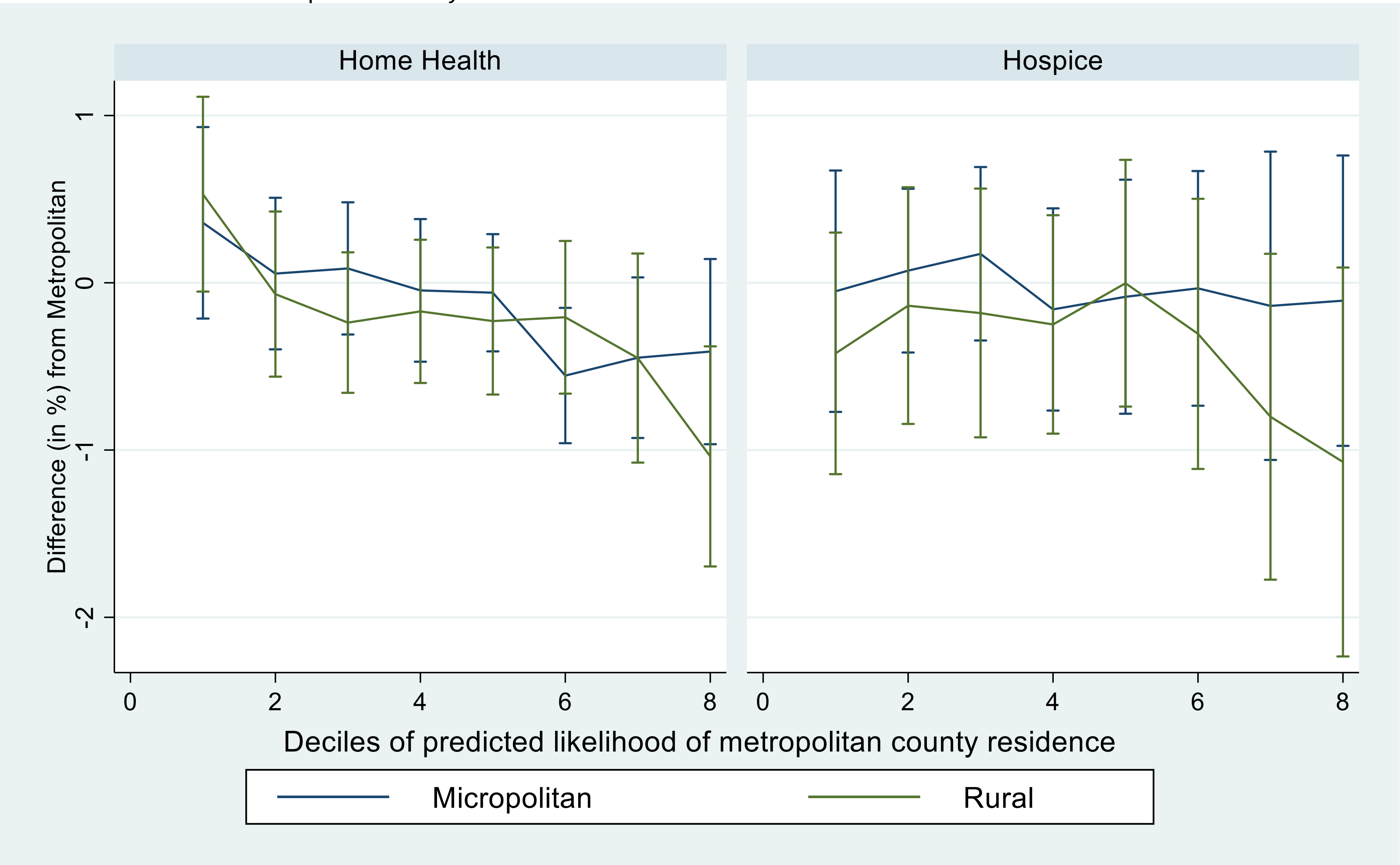

Note: This is the complementary figure of Figure 3 in the manuscript

© 2020 Rahman M et al. *JAMA Network Open.*

eFigure 5. Difference in Outcomes for Medicare Beneficiaries With ADRD Residing in Micropolitan and Rural Counties Relative to Those in Metropolitan Counties by Zip Codes With Different Medicare Advantage (MA) Penetration

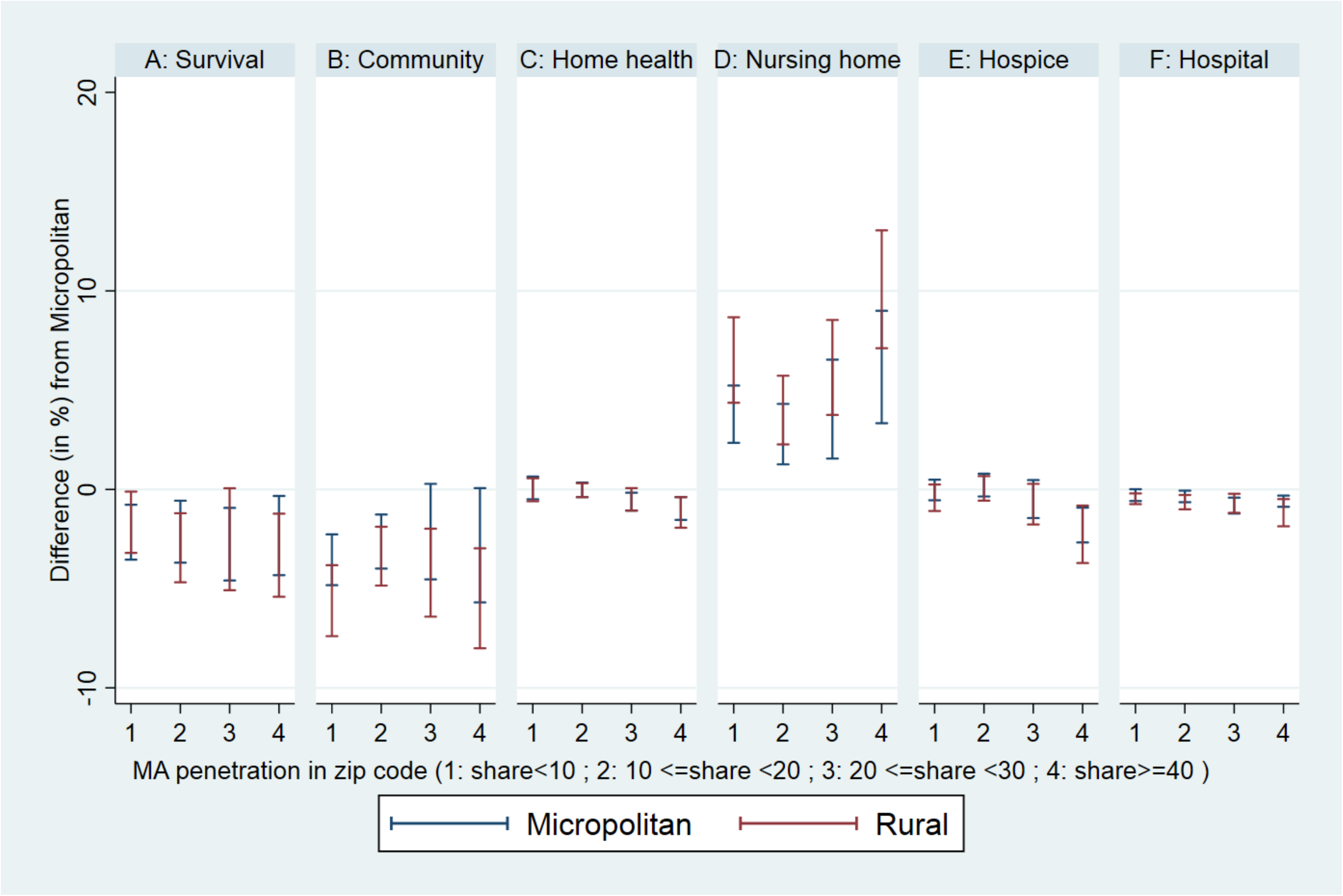

© 2020 Rahman M et al. *JAMA Network Open.*

eFigure 6. Difference in Outcomes for Medicare Beneficiaries With ADRD Residing in Micropolitan and Rural Counties Relative to Those in Metropolitan Counties by Zip Codes With Different Social Deprivation Index

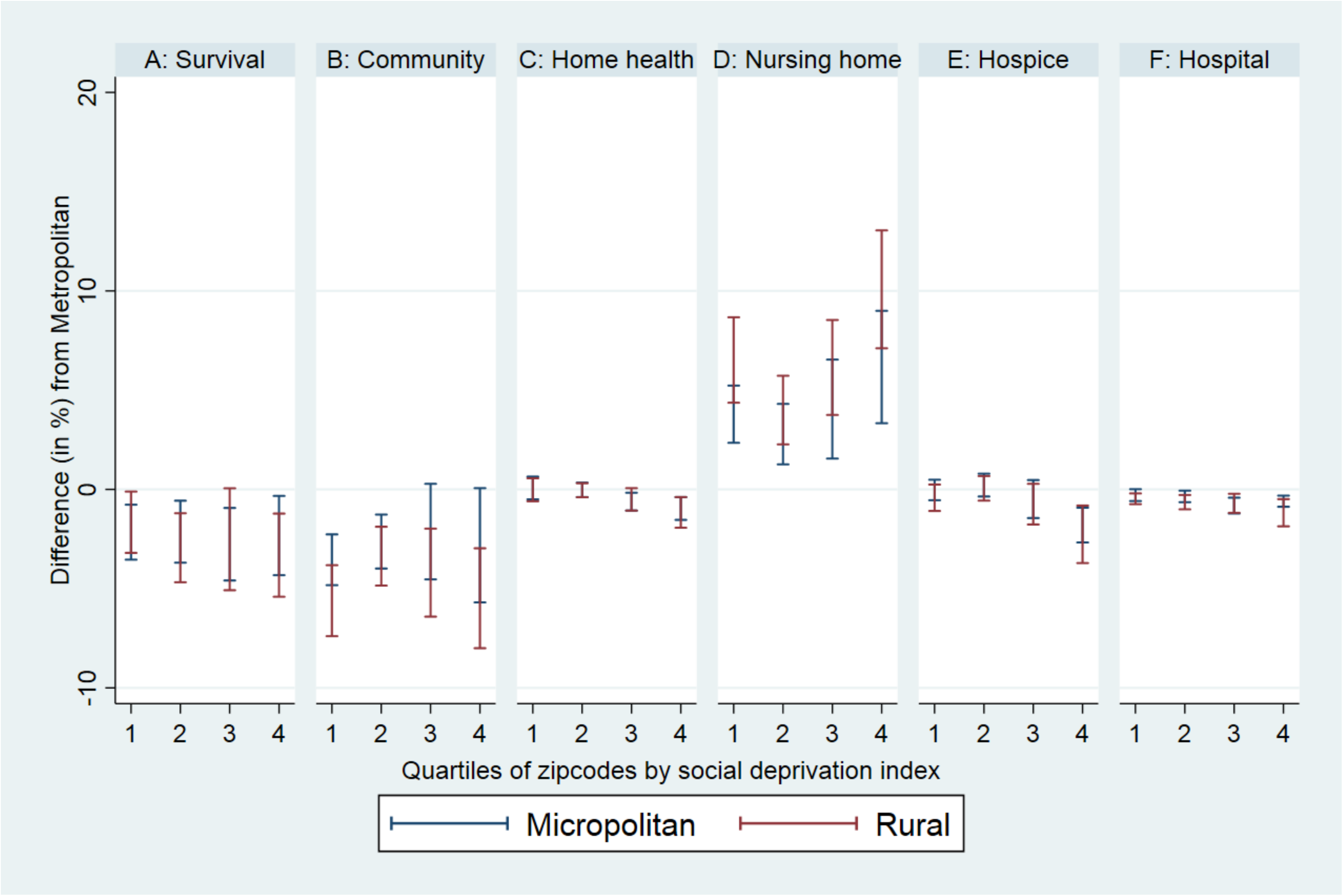

© 2020 Rahman M et al. *JAMA Network Open.*

eTable. Cohort Selection Steps

		Metropolitan	Micropolitan	Rural
Age 65+ Medicare beneficiaries in January 2010 [Mean age]		30,315,353 [75.2]	4,455,756 [74.9]	3,140,177 [74.9]
After exclusion due to Medicare Advantage enrollment, insufficient Medicare enrollment during 3-year look back period and missing covariates [Mean age] (share excluded)		16,154,147 [77.0] (46.7%)	2,960,856 [76.5] (33.5%)	2,172,512 [76.4] (30.8%)
Distribution of ADRD diagnosis	Excluded because Did not have ADRD in 2010 or before (share excluded)	13,809,243 (85.4%)	2,574,940 (87.0%)	2,172,512 (87.0%)
	Excluded because Started 2010 with ADRD diagnosis (share excluded)	1,883,435 (11.65%)	305,672 (10.3%)	222,323 (10.3%)
	Newly diagnosed with ADRD in 2010 (share included)	470,642 (2.9%)	80,244 (2.7%)	59,311 (2.7%)
Final sample after exclusion due to Medicare Advantage enrollment and missing outcome data during the six year follow up period (share excluded)		424,561 (9.8%)	75,001 (6.5%)	55,771 (6.0%)

© 2020 Rahman M et al. *JAMA Network Open.*
